# Rapid Measurement of Cellulose, Hemicellulose, and Lignin Content in *Sargassum horneri* by Near-Infrared Spectroscopy and Characteristic Variables Selection Methods

**DOI:** 10.3390/molecules27020335

**Published:** 2022-01-06

**Authors:** Ning Ai, Yibo Jiang, Sainab Omar, Jiawei Wang, Luyue Xia, Jie Ren

**Affiliations:** 1 College of Biological, Chemical Science and Engineering, Jiaxing University, Jiaxing 314001, China; aining@tsinghua.org.cn; 2 Zhejiang Province Key Laboratory of Biomass Fuel, Hangzhou 310014, China; jyb892902296@sina.com; 3 College of Chemical Engineering, Zhejiang University of Technology, Hangzhou 310014, China; 4 Chemical Engineering and Applied Chemistry, Aston University, Aston Triangle, Birmingham B4 7ET, UK; omars2@aston.ac.uk (S.O.); j.wang23@aston.ac.uk (J.W.)

**Keywords:** near-infrared spectroscopy, rapid measurement, macroalgae, lignocelluloses, variables selection

## Abstract

Near-infrared (NIR) spectroscopy and characteristic variables selection methods were used to develop a quick method for the determination of cellulose, hemicellulose, and lignin contents in *Sargassum horneri*. Calibration models for cellulose, hemicellulose, and lignin in *Sargassum horneri* were established using partial least square regression methods with full variables (full-PLSR). The PLSR calibration models were established by four characteristic variables selection methods, including interval partial least square (iPLS), competitive adaptive reweighted sampling (CARS), correlation coefficient (CC), and genetic algorithm (GA). The results showed that the performance of the four calibration models, namely iPLS-PLSR, CARS-PLSR, CC-PLSR, and GA-PLSR, was better than the full-PLSR calibration model. The iPLS method was best in the performance of the models. For iPLS-PLSR, the determination coefficient (R^2^), root mean square error (RMSE), and residual predictive deviation (RPD) of the prediction set were as follows: 0.8955, 0.8232%, and 3.0934 for cellulose, 0.8669, 0.4697%, and 2.7406 for hemicellulose, and 0.7307, 0.7533%, and 1.9272 for lignin, respectively. These findings indicate that the NIR calibration models can be used to predict cellulose, hemicellulose, and lignin contents in *Sargassum horneri* quickly and accurately.

## 1. Introduction

Fossil fuels such as coal, oil, and natural gas have facilitated the economic, social, and technological development of countries around the globe. As development continues, the demands for fossil fuels are steadily increasing. However, the excessive consumption of fossil fuels with limited reserves has instigated an energy crisis and is also accompanied by air pollution, climate change, and other environmental issues. As a result, an international consensus to transform the structure of global energy and introduce renewable energy systems was established [1].

Up to now, the lignocellulosic material from *Sargassum horneri* has been developed and applied in many areas, such as polysaccharides from marine algae [2], cement-bonded lignocellulosic fiber composites used in construction companies [3], and insulation panels from multilayered coir long and short fiber reinforced phenol formaldehyde polymeric (PF) resin [4]. What is more, several forms of the nanocellulose have been reported as building blocks for producing hydro- and aerogels [5]. Hence, the utilization of *Sargassum horneri* has attracted the interest of academics. The decomposition of the cell wall, hydrolysis of monosaccharides, and biological fermentation are the three main steps in the conversion of biomass to biofuel. In this process, the Ni/CNT catalysts [6], Ni/Carbon nanotubes functionalized catalysts [7], and 2D Mxene nano-sheets (Ti_3_C_2_T_x_) [8] are the possible topic catalysis. Lignocelluloses (cellulose, hemicellulose, and lignin), which constitute the cell wall of plants and its physical structure, influence the process of cell wall decomposition [9,10]. After hydrolysis, lignocelluloses are converted into various monosaccharides needed for fermentation, and the content of lignocellulose directly affects the yield of biofuels [11,12,13,14]. Thus, the content of lignocellulose in biomass is a vital quality control indicator in biofuel production.

The analytical methods of lignocellulose can be divided into chemical analysis and instrumental analysis. Generally, the chemical analysis methods include Van Soest [15], Klason [16,17,18], and oxidase-DNS [19,20]. Instrumental analysis methods such as gas chromatography (GC) and high-performance liquid chromatography (HPLC) [21,22] are more accurate than chemical analysis. Determining the cellulose, hemicellulose, and lignin contents in biomass using chemical or instrumental analysis is laborious, time-consuming, and expensive. As a result, it is necessary to develop a fast, simple, precise, and cost-effective method for quantifying lignocellulose in biomass.

With the rapid development of chemometrics, the main disadvantages such as low absorptance, serious overlap, and weak characteristics in the application of near-infrared (NIR) spectroscopy have been overcome. The NIR spectroscopy analytical technique has been widely used for the prediction of quality parameters in the fields of food, agriculture, forestry, and energy due to the advantages of rapid analysis, concise operation, and accurate and nondestructive testing [23,24,25,26,27,28]. NIR spectroscopy records the spectral bands that correspond to C-H, O-H, N-H vibrations, which are abundant in lignocellulose; this is the theoretical basis for using NIR spectroscopy to determine the lignocellulose content in biomass. Hayes et al. [29,30] predicted the lignocellulose components of wet Miscanthus samples and peat samples with NIR spectroscopy accurately. Partial least square regression (PLSR), least squares support vector machine regression (LSSVR), and radial basis function neural network (RBF NN) have been used to establish the calibration models of Miscanthus sinensis lignocellulose [31].

Currently, NIR spectroscopy models are usually established for the determination of terrestrial biomass. However, the conversion processes of terrestrial biomass, such as corn and sugarcane, are expensive and influence the stability of the food supply. Marine biomass, such as macroalgae, does not require arable land and can be grown in many diverse environments, and it can be planted in different seasons to ensure a steady supply of biomass throughout the year. Hence, environmental pollution can be recovered to a certain extent. Most importantly, great advances have been made in the conversion of macroalgae to biofuel. Therefore, the technology and systems are in place for the effective utilization of biomass energy of macroalgae [32,33].

The expected use of algae biomass is to become one of the ideal biomass alternative resources, such as biogas, ethanol, and other biomass liquid fuels produced by algae fermentation. However, there is little information available on the use of NIR spectroscopy for rapid detection of the lignocellulose content in macroalgae. Moreover, the NIR calibration models that are established based on terrestrial biomass are difficult to apply to the analysis of marine biomass due to differences in biomass composition and content [34]. In order to achieve this purpose, it is necessary to quickly determine the content of cellulose, hemicellulose, or lignin in biomass energy raw materials. Hence, the present study established NIR calibration models with the PLSR method for the simultaneous determination of the content of cellulose, hemicellulose, and lignin in *Sargassum horneri*. The characteristic variable selection methods included the interval partial least square method (iPLS), competitive adaptive reweighted sampling (CARS), correlation coefficient method (CC), and genetic algorithm (GA). Compared with the conventional analysis method, the total determination time of a single sample by the near-infrared spectroscopy method would be far less if the accuracy of established NIR calibration models with the PLSR method is acceptable.

## 2. Materials and Methods

### 2.1. Sample Collection and Preparation

Seventy-four *Sargassum horneri* samples were collected from Ningbo, Zhejiang Province, China (E120° 43.428′, N281° 1.938′). All samples were washed with clean water to remove the sediment and saline matter on the surface. The cleaned samples were dried in the sun for 16 h and in the oven at 105 °C for 12 h. All samples were pulverized and sieved through a 60-mesh sieve. The collected powder was stored in a desiccator.

### 2.2. Measurement of Cellulose, Hemicellulose, and Lignin

The contents of cellulose and hemicellulose in the *Sargassum horneri* samples were measured using the Van Soest analytical procedure [15], while the contents of lignin were measured using the National Renewable Energy Laboratory (NREL) method [35]. The contents of lignin were a combination of both acid-insoluble and acid-soluble lignin. The 210 nm wavelength was used for the UV-Vis measurement because acid-soluble lignin was included. All reagents used in this study were of analytical grade. The relative errors of the chemical measurements were maintained below ±5%.

### 2.3. NIR Spectra Acquisition

The NIR spectra of the *Sargassum horneri* samples were collected in the diffuse reflection mode by a Fourier transformation infrared spectrometer (Nicolet iS10, Thermo Fisher Scientific, Waltham, MA, USA). Four grams of sample powder was placed in a rotatable sample cup. Spectral acquisition was carried out at an ambient temperature of 25 °C and relative humidity of 30% using air as the reference standard. The NIR spectra of background were collected before the collection of the *Sargassum horneri* samples, and the background subtraction was automatically performed after the collection of the *Sargassum horneri* samples. The spectral scanning range was from 12,000 cm^−1^ to 4000 cm^−1^. Scanning was executed 64 times. Each sample was scanned three times, and the average spectrum was regarded as the sample spectrum. A total of 8298 data points per spectrum were obtained by measuring the spectrum at intervals of 0.964 cm^−1^.

### 2.4. Multivariate Data Analysis

#### 2.4.1. Data Partition

Selecting representative samples in the process of establishing the NIR spectroscopy model could improve the speed and accuracy of modeling and facilitate updating and maintenance of the model. The Kennard-Stone (K-S) method [36] was used to divide the original samples into two groups: 48 samples were used as a calibration set and the remaining 26 samples as a prediction set.

#### 2.4.2. Spectral Pretreatment

Background noise and light scattering of the samples are the main factors causing error during NIR spectral acquisition. Spectral pretreatment methods are necessary to eliminate noise in the spectra and enhance effective spectral information. This improves the stability and prediction accuracy of the NIR spectroscopy models. The pretreatment methods used in NIR spectroscopy included the Savitzky-Golay (SG) smoothing method, Savitzky-Golay derivative method, standard normal variate transformation (SNV), and multiplicative scatter correction (MSC) [37,38].

The SG method can be used to improve the signal-noise ratio (SNR) of spectra by removing irregular random noise. Moreover, derivative methods such as Savitzky-Golay first derivative (SG+1st) and Savitzky-Golay second derivative (SG+2nd) can effectively remove the baselines and enhance the identification of overlapping peaks. SNV and MSC can also be used to eliminate the influence of particle size, surface scattering, and optical path change in NIR spectra. In the present study, spectral pretreatment methods, including SG, SG+1st, SG+2nd, SNV, and MSC, were investigated.

#### 2.4.3. Selection of Characteristic Variables

NIR spectroscopy models that cover the whole spectral band can provide complete component information. However, not all the component information retained is necessary. The excess data cause difficulties in distinguishing specific component information of the model. Thus, the spectral band range can be reasonably reduced to eliminate the irrelevant or non-linear variables in the spectrum and concentrate the representative characteristic variables. This improves stability and prediction accuracy and accelerates the calculation speed of the model [39,40].

Characteristic variables selection methods, including interval partial least squares (iPLS) [41], competitive adaptive reweighted sampling (CARS) [42], correlation coefficient (CC) [43], and genetic algorithm (GA) [44,45], were applied.

#### 2.4.4. Development and Evaluation of NIR Models

All NIR spectroscopy models were established based on the PLSR method. The performances of the established models were evaluated by the following criteria: coefficient of determination (R^2^), root mean square error (RMSE), and residual predictive deviation (RPD). R^2^ was used to evaluate the linear correlation between the predicted value of the model and the chemical analysis value. RMSE reflected the deviation between the predicted value of the model and the chemical analysis value. A lower value of RMSE indicates higher prediction accuracy. RPD is defined as the ratio of the standard deviation of the calibration set to the RMSE of the prediction set and was calculated to assess the prediction performance of the model. Generally, an RPD value greater than 1.5 indicates that the model could be used for rough predictions. A model with an RPD value between 2.0 and 2.5 suggests good prediction performance, and the model could be used for high-precision prediction when RPD is greater than 3.0.

### 2.5. Software

OMNIC software (version 8.3, Thermo Fisher Scientific, Waltham, MA, USA) was used for the collection of NIR spectra. MATLAB software (version 2016a, MathWorks, Natick, MA, USA) was used for spectral pretreatment, variable selection, and PLSR modeling.

## 3. Results and Discussion

### 3.1. NIR Spectral Features

**Figure 1:**
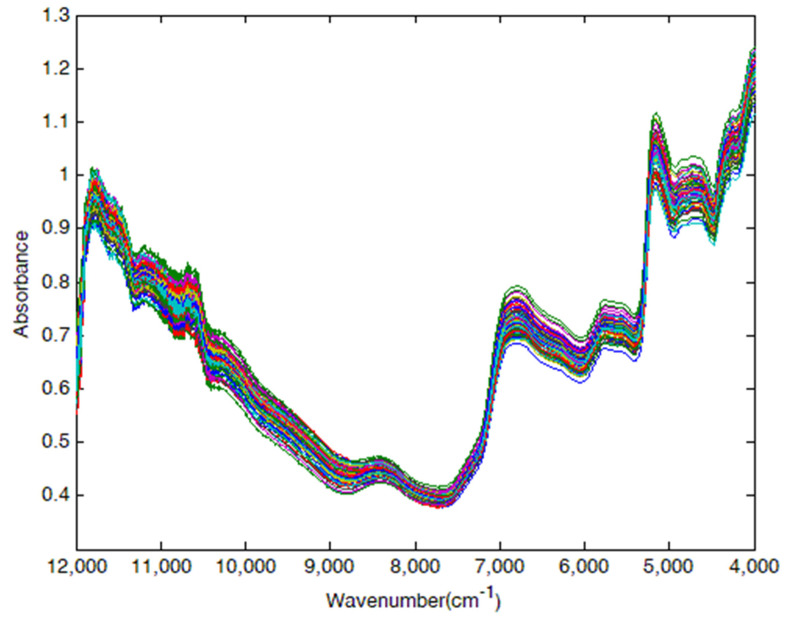
Raw NIR spectra of *Sargassum horneri* samples.

Figure 1 shows the raw NIR spectra of the 74 *Sargassum horneri* samples in the range of 12,000 to 4000 cm^−1^. The peak at 11,800 cm^−1^ was assigned to the 4ν stretching vibration of N-H, while the peak located at 11,000 cm^−1^ was associated with the 4ν stretching vibration of C-H in methyl or methylene. The peak at 10,400 cm^−1^ was attributed to the 3ν stretching vibration of free hydroxyl group, and the signals at 8400 cm^−1^ and 6900 cm^−1^ were ascribed to the C-H in methyl or methylene, the former of which belonged to 3ν stretching vibration and the latter may be due to the combination of 2ν stretching vibration and the ν bending vibration. The peak near 5800 cm^−1^ originated from the 2ν stretching vibration of the methyl, methylene, or mercapto group, and the peak observed at 5000 cm^−1^ could be related to the 3ν bending vibration as well as the combination of 2ν bending vibration and v stretching vibration in the free hydroxyl groups. The raw NIR spectra presented many disadvantages such as abundant spike peaks, serious overlap, and weak characteristics. As a result, it was difficult to directly establish a link between the spectra and the target components based on the information on peak position, peak strength, and peak shape.

Yeh et al. [34] identified the peak at 5980 cm^−1^ as a characteristic variable for cellulose, and three wavelengths, including 7320 cm^−1^, 6983 cm^−1^, and 6297 cm^−1^, significantly and positively correlated with lignin. Nabavi et al. [46] established a model based on NIR spectra to predict the properties of loblolly pine tracheid. The authors found the absorbance wavenumber range 8621 cm^−1^ to 4803 cm^−1^ was highly correlated to cellulose. Similarly, the peaks at 4303 cm^−1^ and 7062 cm^−1^ were useful for the establishment of hemicellulose and lignin in the calibration models, respectively. Based on the regression coefficient, Zhang et al. [43] established calibration models of cellulose (7000 to 5500 cm^−1^) and hemicellulose (7500 to 4000 cm^−1^). The characteristic variables, including 5934 cm^−1^, 5980 cm^−1^, 5888 cm^−1^, 4682 cm^−1^, 4410 cm^−1^, 4417 cm^−1^, and 4196 cm^−1^, were positively related with lignin in the study by Fahey et al. [47].

Among the above-mentioned characteristic variables, the peaks at 8400 cm^−1^, 6900 cm^−1^, 5800 cm^−1^, and 5000 cm^−1^ in NIR spectroscopy were dovetailed with references. By selecting these characteristic variables, better calibration models for cellulose, hemicellulose, and lignin in *Sargassum horneri* were established.

#### 3.1.1. Division of Calibration and Prediction Set

Seventy-four samples of *Sargassum horneri* were divided into the calibration set or prediction set by the K-S method, and the proportion of division was approximately 2:1. The samples in the calibration set were used to establish the model, while the samples in the prediction set were used to test the accuracy of the established model. The statistical data of total sets, calibration sets, and prediction sets with regard to the concentration of lignocellulose in *Sargassum horneri* are listed in Table 1.

Table 1 shows that the content range region, mean, and standard deviation of data of the total sets, calibration sets, and prediction sets are consistent after division, and the coefficient of variation was less than 10. This result indicates that the samples were clustered, so the division of the samples was appropriate for NIR spectra analysis.

Table 2 shows the cellulose, hemicellulose, and lignin contents in *Sargassum horneri* and other plant fibers. It can be found that the lignocellulose content of *Sargassum horneri* is similar to that of the terrestrial biomass. Hence, the utilization of *Sargassum horneri* is promising.

#### 3.1.2. Spectral Pretreatment

SG, SG+1st, SG+2nd, SNV, and MSC were applied to the pretreatment of the raw NIR spectra, and five models were established. Table 3 shows the performance indices of the models. The optimum method for cellulose, hemicellulose, and lignin models in *Sargassum horneri* were SG with a window width of 270 cm^−1^, SG+1st with a window width of 357 cm^−1^, and SG+2nd with a window width of 289 cm^−1^, respectively. According to Table 3, the optimal selection results of spectral pretreatments for cellulose, hemicellulose, and lignin models were different.

#### 3.1.3. Performance of Multivariate Calibration Models

In this study, iPLS, CARS, CC, and GA were performed to establish calibration models with good predictive performance. Four different regression models, namely, iPLS-PLSR, CARS-PLSR, CC-PLSR, and GA-PLSR, were established. Full-PLSR models indicated that the PLSR model was constructed on the basis of a full spectrum. The comparative performances of the five regression models are presented in Table 4.

### 3.2. Results of the Full-PLSR Model

As shown in Table 4. the RPD values for the full-PLSR models of cellulose, hemicellulose, and lignin were 1.6139, 1.5004, and 1.1221, respectively. This result indicates that the full-PLSR model achieved a rough prediction for cellulose and hemicellulose content. The performance of the model for determining the lignin content was relatively inferior. The results were mainly attributed to the effect of non-target components in the NIR spectra. The presence of non-target components made it difficult to identify specific target components, which decreased the accuracy of the models. Thus, the prediction quality of the full-PLSR models required further improvement with the aid of variable selection methods.

### 3.3. Results of the iPLS-PLSR Model

**Figure 2:**
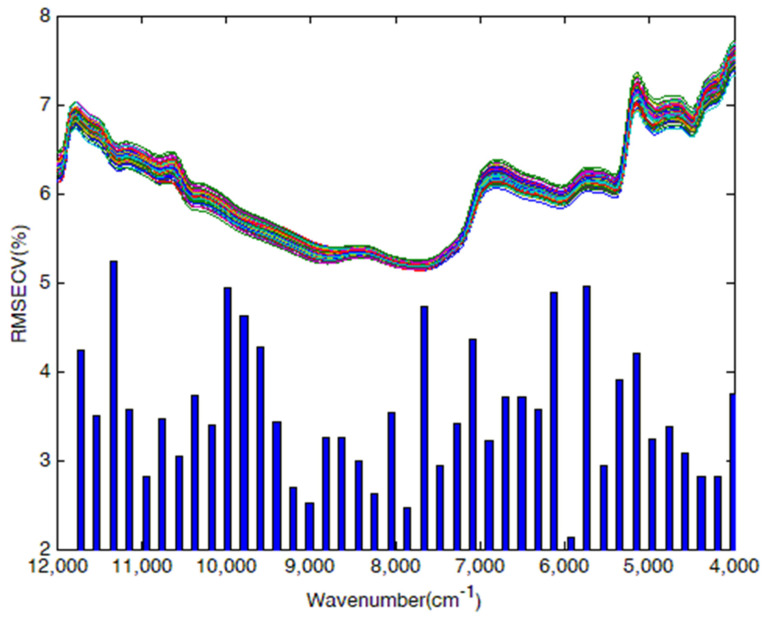
Plots of characteristic variable selection based on iPLS for cellulose in *Sargassum horneri*.

By developing the interval partial least square method (iPLS), the whole wavelengths were divided into 69 sub-intervals with a width of 116 cm^−1^, and the significance of each sub-interval was evaluated by the leave-one-out cross-calibration method. Taking cellulose as an example, Figure 2 shows the RMSECV values of sub-intervals in the process of characteristic variables selection by iPLS. The sub-intervals at 10,940 cm^−1^, 9015 cm^−1^, 7858 cm^−1^, 5929 cm^−1^, 5543 cm^−1^, 4386 cm^−1^, and 4193 cm^−1^ had lower RMSECV values, and these sub-intervals were expanded bidirectionally with a step length of 3.9 cm^−1^. The expanded sub-intervals were randomly merged, and cross-validation was used to select the interval with the lowest RMSECV value. At this time, the number of characteristic variables was 1540.

Finally, the CV characteristic variables 1540, 1935, and 1665 were selected to establish the NIR spectroscopy models for cellulose, hemicellulose, and lignin in *Sargassum horneri*, which were less than 8298 variables in the whole wavelengths. The optimum characteristic variables selected for cellulose, hemicellulose, and lignin were 5484 to 4000 cm^−1^, 5865 to 4000 cm^−1^, and the combination of 7104 to 6560 cm^−1^ and 5060 to 4000 cm^−1^, respectively.

According to Table 4, the performance of the iPLS-PLSR models was much better than that of the full-PLSR models. The $R_{P}^{2}$ improved from 0.6161, 0.5558, and 0.2058 to 0.8955, 0.8669, and 0.7307, respectively. The RMSEP (%) decreased from 1.3833, 0.9735, and 1.3833 to 0.8232, 0.4697, and 0.7533, respectively. The RPD increased from 1.6139, 1.5004, and 1.1221 to 3.0934, 2.7406, and 1.9272, respectively.

### 3.4. Results of the CARS-PLSR Model

**Figure 3:**
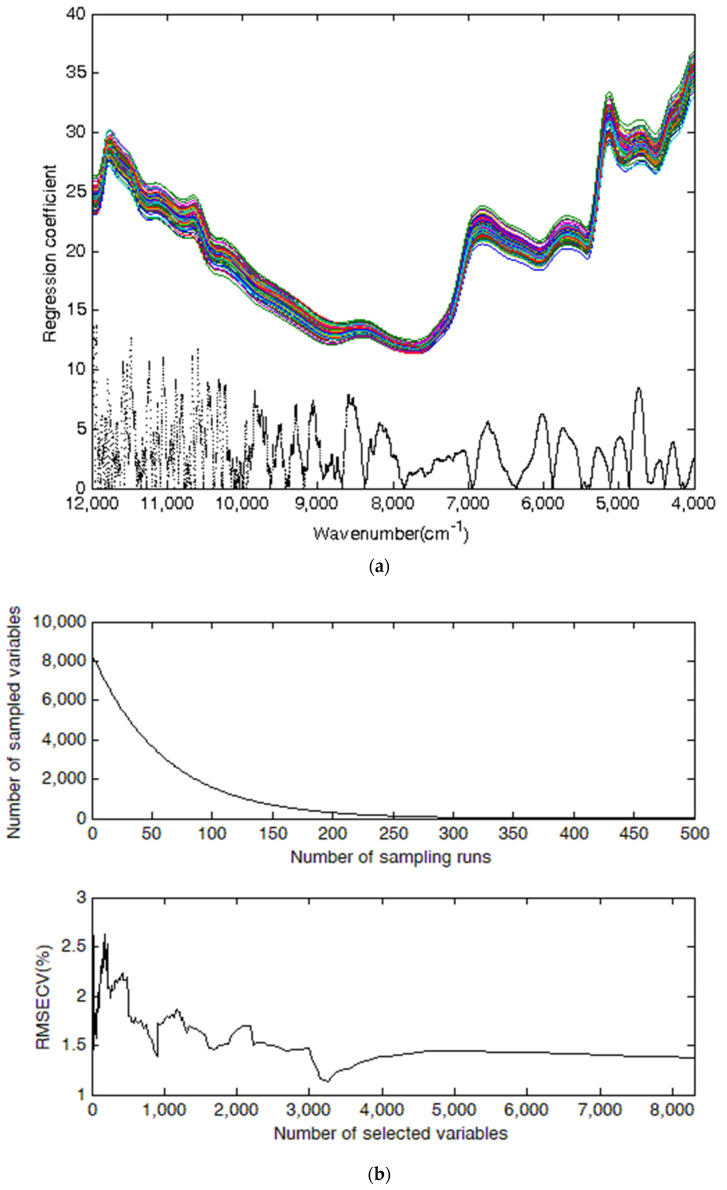
Plots of characteristic variable selection based on CARS for cellulose in *Sargassum horneri*. Plot (**a**), ((**b**) upper), and ((**b**) lower) show the regression coefficient, number of sampled variables, and RMSECV value, respectively.

In model development by competitive adaptive reweighted sampling (CARS), taking cellulose as an example, Figure 3 shows the process of selecting characteristic variables based on CARS for cellulose. Figure 3a, Figure 3b (upper), and Figure 3b (lower) are used to illustrate the change in the regression coefficient, number of sampled variables, and RMSECV in the process of selecting characteristic variables, respectively. Figure 3a indicates that the sub-intervals in 12,000 to 10,000 cm^−1^ or around 9090 cm^−1^, 8553 cm^−1^, 8195 cm^−1^, 6721 cm^−1^, 6010 cm^−1^, and 4745 cm^−1^ had high regression coefficient values. Higher values of the absolute regression coefficient suggest a higher probability that the corresponding variables should be selected. Figure 3b (upper) shows the variation of the sampling number of variables with the number of CARS runs, which decreased rapidly with the increase of the number of runs then tended to be flat. In Figure 3b (lower), the value of RMSECV decreased fast at first and then increased slowly with the continuous growth in variables. The minimal RMSECV was obtained when the number of selected variables was 3261.

Finally, 3261, 6461, and 3328 variables were selected to establish the NIR spectroscopy models of cellulose, hemicellulose, and lignin, respectively, which were less than 8298 variables in the whole wavelengths.

According to Table 4, the performance of the CARS-PLSR models was better than the full-PLSR models. The $R_{P}^{2}$ improved from 0.6161, 0.5558, and 0.2058 to 0.7742, 0.6962, and 0.4119, respectively. The RMSEP (%) decreased from 1.3833, 0.9735, and 1.3833 to 1.0637, 0.9624, and 0.9015, respectively. The RPD increased from 1.6139, 1.5004, and 1.1221 to 2.1043, 1.8143, and 1.3040, respectively.

### 3.5. Results of the CC-PLSR Model

**Figure 4:**
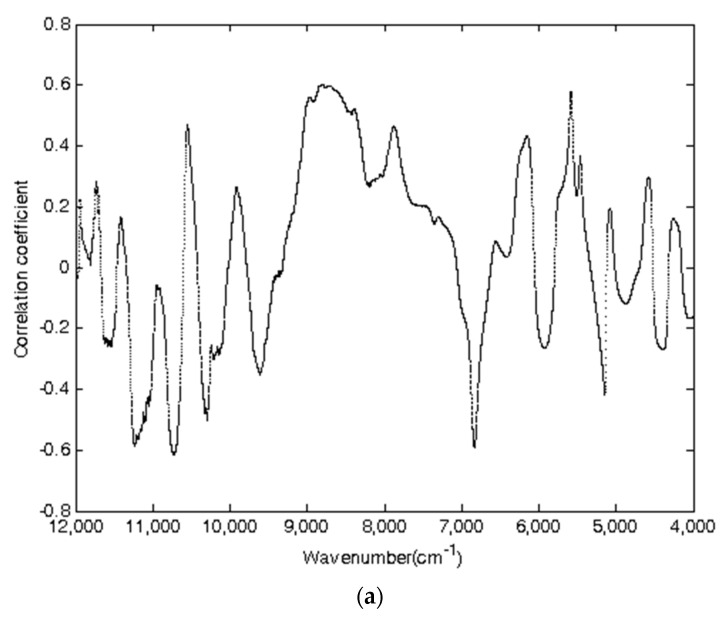

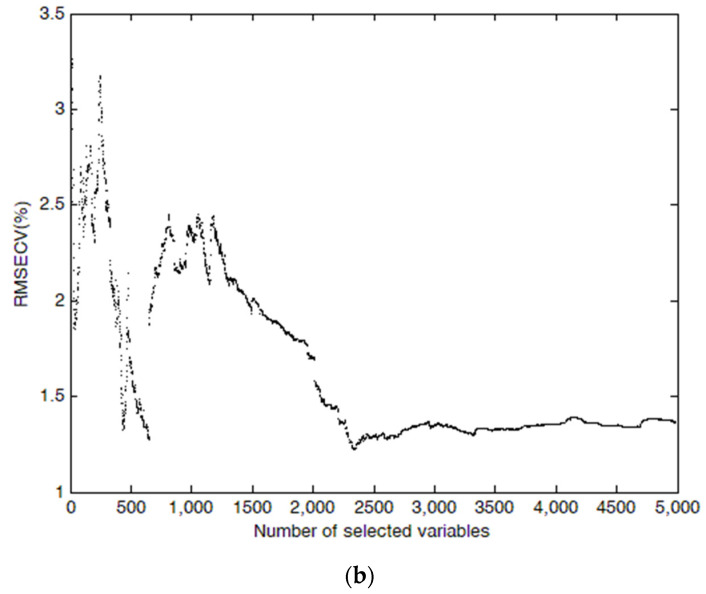
Plots of characteristic variable selection based on CC for cellulose in *Sargassum horneri*. Plots (**a**,**b**) show the correlation coefficient and RMSECV value, respectively.

In model development by the correlation coefficient method (CC), taking cellulose as an example, Figure 4 shows the process of selecting characteristic variables based on CC for cellulose. The distribution of correlation coefficient based on the whole wavelengths and the intervals covering 11,260 to 10,670 cm^−1^, 9000 to 7700 cm^−1^, and 5640 to 5558 cm^−1^ had higher values of the correlation coefficient. Figure 4b describes the value of RMSECV with an increase in the selected variables, where the variable with a higher value of the absolute correlation coefficient would be selected at first. Although the value of RMSECV was unsteady when the number of variables was less than 790, the tendency of RMSECV decreased rapidly in the beginning and then increased slowly. The value of RMSECV was steady when the number of variables was more than 3000. The minimal RMSECV was obtained when the number of selected variables was 2485 (Figure 4b).

Finally, the CV characteristic variables 2485, 1705, and 2264 were selected to establish the NIR spectroscopy models of cellulose, hemicellulose, and lignin, respectively, which were less than 8298 variables in the whole wavelengths.

According to Table 4, the performance of the CC-PLSR models was slightly better than that of the full-PLSR models. The $R_{P}^{2}$ improved from 0.6161, 0.5558, and 0.2058 to 0.7353, 0.6746, and 0.4460, respectively. The RMSEP (%) decreased from 1.3833, 0.9735, and 1.3833 to 1.2988, 0.7689, and 1.1685, respectively. The RPD increased from 1.6139, 1.5004, and 1.1221 to 1.9437, 1.7532, and 1.3435, respectively.

### 3.6. Results of the GA-PLSR Model

In model development by the genetic algorithm (GA), the parameters were set as follows: the iterations were 200, the initial population was 20, the crossover probability was 0.7, and the mutation probability was 0.05. The RMSECV was selected as the fitness function, and the leave-one-out cross-calibration method was used to search the characteristic variables with the lowest value of RMSECV. Considering that GA is mainly a stochastic algorithm whose results varied in different experimental periods, the genetic algorithm was calculated repeatedly, and the frequency of selection of each variable was recorded in multiple runs.

**Figure 5:**
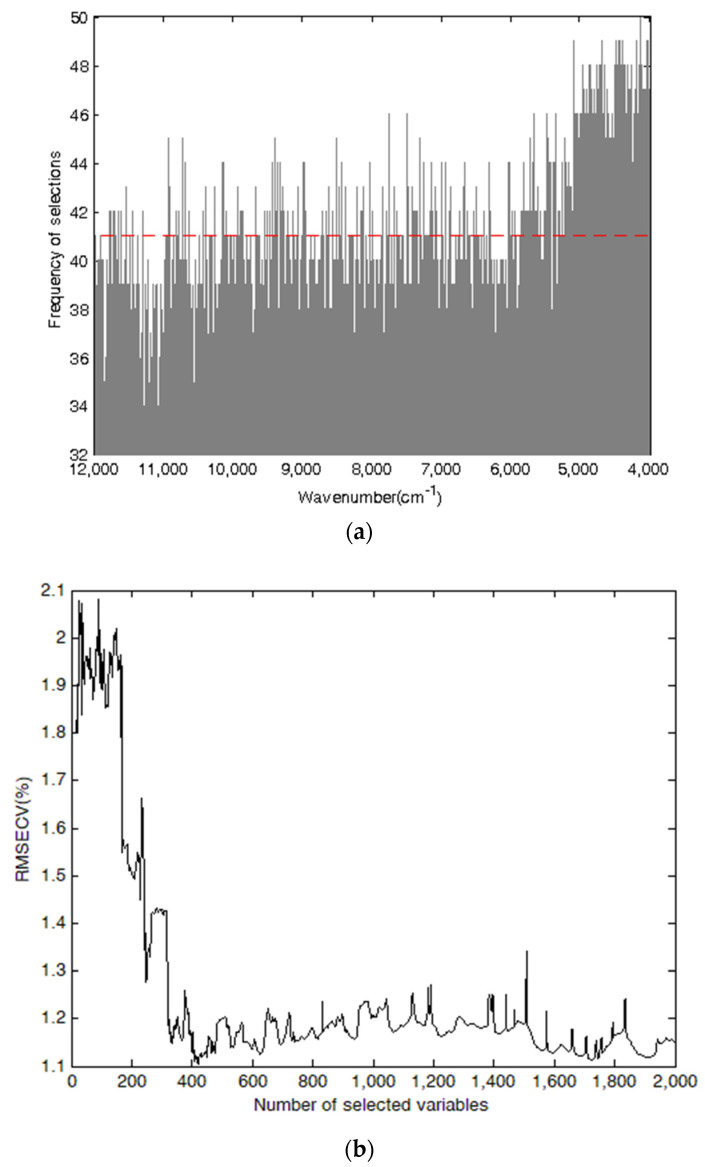
Plots of characteristic variable selection based on GA for cellulose in *Sargassum horneri*. Plots (**a**,**b**) show the variable frequency and RMSECV value, respectively.

Taking cellulose as an example, Figure 5 shows the process of selecting characteristic variables based on GA for cellulose. Figure 5a demonstrates the frequencies of each variable selected after 50 runs by GA. The variables with higher frequencies were mainly distributed in the region of 5229 to 4000 cm^−1^, especially in the intervals covering 5028 to 4668 cm^−1^ and 4492 to 4064 cm^−1^. The higher the frequency, the more probable the corresponding variable would be selected. The cross-validation method was also used to evaluate the performance of the model. Figure 5b shows the change in the RMSECV value with the increasing number of selected variables. The value of RMSECV decreased rapidly as the number of variables increased from 1 to 421, which was ascribed to the variables including vital information until it arrived at a minimum and held a relatively constant level. The lowest RMSECV value was found in 421 variables, as shown by a red dashed line in Figure 5a.

Finally, 421, 731, and 899 variables were selected to establish the NIR spectroscopy models of hemicellulose and lignin, respectively. The number of selected variables was all less than 8298 variables in the whole wavelengths.

According to Table 4, the performance of the GA-PLSR models was slightly better than the full-PLSR models. The $R_{P}^{2}$ improved from 0.6161, 0.5558, and 0.2058 to 0.7418, 0.7201, and 0.3660, respectively. The RMSEP (%) decreased from 1.3833, 0.9735, and 1.3833 to 1.1506, 0.8029, and 1.3635, respectively. The RPD increased from 1.6139, 1.5004, and 1.1221 to 1.9680, 1.8902, and 1.5796, respectively.

### 3.7. Comparison of the Results by Four Variable Selection Methods

Table 4 shows that all four variable selection methods performed better than the original full-PLSR model. A clear ranking was noticed as follows: iPLS > CARS > GA ≈ CC for cellulose, iPLS > GA ≈ CARS > CC for hemicellulose, and iPLS > GA > CC ≈ CARS for lignin. It was obvious that iPLS was the best method for selecting characteristic variables for cellulose, hemicellulose, and lignin, while the CC method was difficult to provide effective improvements due to the synergistic interaction in variables and the nonlinearity of the spectra. Selecting useful variables from the full spectra is very important for the improvement of the calibration model. Variable selection methods reduce the number of variables and the complexity of models and improve the quality and the robustness of the calibration models.

The regions of characteristic variables optimized for models were 5484 to 4000 cm^−1^ (cellulose), 5865 to 4000 cm^−1^ (hemicellulose), and the combination of 7104 to 6560 cm^−1^ and 5060 to 4000 cm^−1^ (lignin), respectively. The 3ν bending vibration, as well as the combination of 2ν bending vibration and v stretching vibration in the free hydroxyl groups, were included in these regions. In particular, the 2ν stretching vibration of methyl and methylene contributed to the hemicellulose model, and the 2ν stretching vibration and the ν bending vibration of C-H in methyl or methylene were used to establish the lignin model.

**Figure 6:**
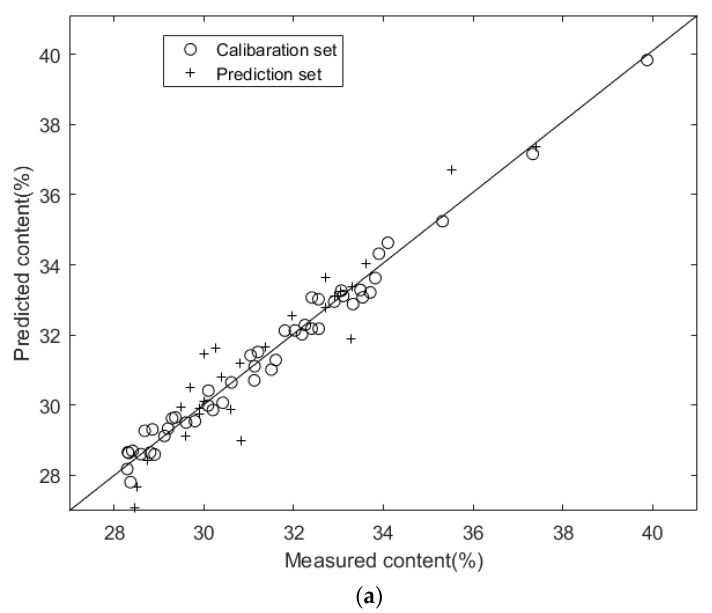

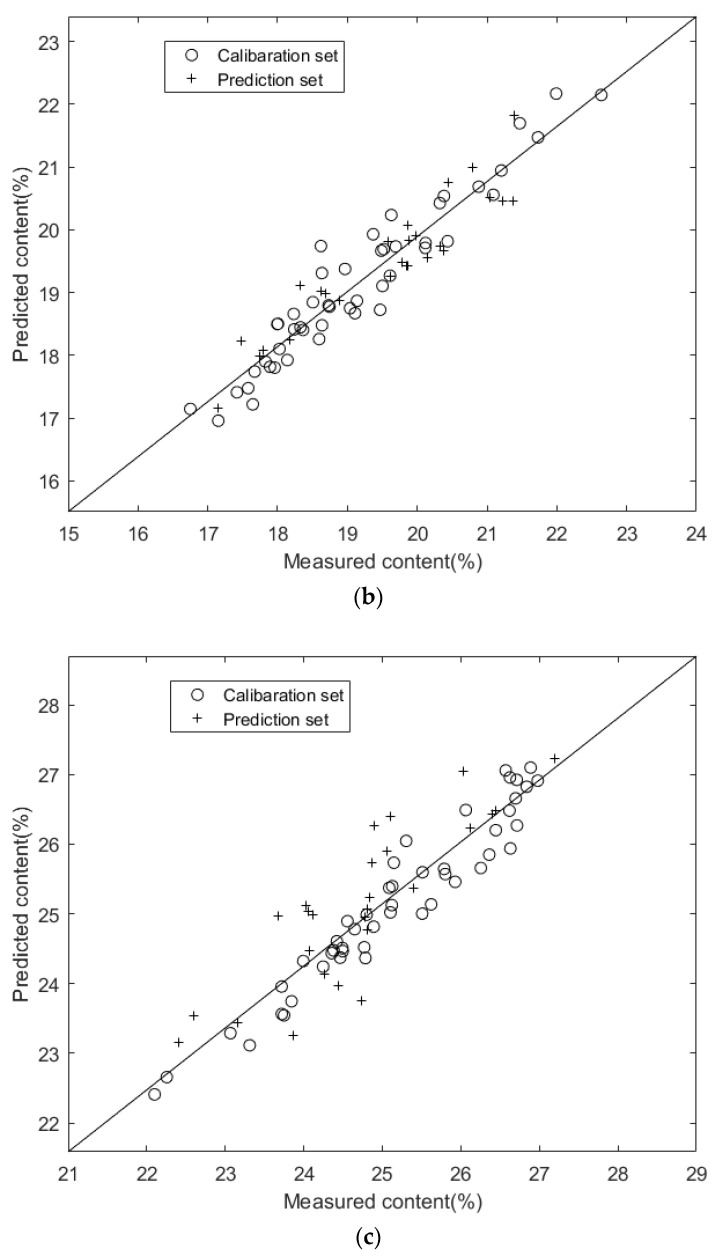
Scatter plots of reference measurements and NIR predictions using iPLS-PLSR models for cellulose (**a**), hemicellulose (**b**), and lignin (**c**) in *Sargassum horneri*.

Figure 6 shows the scatter plots of reference measurements and NIR predictions for the contents of hemicellulose, cellulose, and lignin in *Sargassum horneri* using the iPLS-PLSR models. All data points clustered closely to the diagonal lines, indicating that the iPLS-PLSR models performed better in relating the predicted and reference values.

To date, the rapid measurement of cellulose, hemicellulose, and lignin content in terrestrial biomass by near-infrared spectroscopy has been thoroughly researched, while the *Sargassum horneri* has not. Table 5 shows the results of the near-infrared spectroscopy measurement about terrestrial biomass and *Sargassum horneri*. Compared with the results from other research about terrestrial biomass, the accuracy of the results on *Sargassum horneri* is close to that of the results on terrestrial biomass.

## 4. Conclusions

In this article, the feasibility of the rapid measurement of cellulose, hemicellulose, and lignin in *Sargassum horneri* by near-infrared spectroscopy was verified. Four characteristic variables selection methods were used to improve the performance of models, and the iPLS method proved to be best. The RPD value of the iPLS-PLSR models for cellulose, hemicellulose, and lignin in *Sargassum horneri* were 3.0934, 2.7406, and 1.9272, respectively. For *Sargassum horneri*, iPLS is indeed the best among the four feature selection methods.

However, further research is needed, as the development of these NIR calibration models represents only an initial step, the research object is too narrow, and the number of samples is insufficient. In order to enhance the application scope of the research results, it is necessary not only to collect samples of copper algae from different sea areas but also to collect other kinds of large algae. The near-infrared spectra and lignocellulose content data of these samples were collected to modify or improve the current calibration model so as to establish a universal three-group correction model of lignocellulose in large seaweeds.

The research work in this paper provides a reference for the rapid quality evaluation and control of large seaweeds. The proposed near-infrared spectroscopy analysis method can also be applied to quality analysis in the fields of medicine, agriculture, and food and can be applied to other large algae and lignocellulosic wastes.

## Figures and Tables

**Table 1:** Reference measurement results of samples in the calibration and prediction sets (lignocellulose in *Sargassum horneri*).

	Range	Mean	SD ^1^	CV ^2^ (%)
Total sets (n = 74)				
Cellulose (%)	28.29–39.88	31.37	2.3604	7.5237
Hemicellulose (%)	16.75–22.66	19.28	1.3157	6.8229
Lignin (%)	22.10–27.20	27.04	1.2092	4.4725
Calibration sets (n = 48)				
Cellulose (%)	28.29–39.88	31.39	2.4720	7.8760
Hemicellulose (%)	16.75–22.64	19.14	1.3463	7.0349
Lignin (%)	22.10–26.98	25.14	1.2348	4.9126
Prediction sets (n = 26)				
Cellulose (%)	28.46–37.40	31.35	2.1860	6.9738
Hemicellulose (%)	17.14–21.39	19.55	1.2371	6.3266
Lignin (%)	22.41–27.20	24.70	1.1416	4.6220

^1^ Standard deviation; ^2^ coefficient of variation ((SD/mean) × 100).

**Table 2:** Cellulose, hemicellulose, and lignin contents in *Sargassum horneri* and other plant fibers.

	Cellulose (%)	Hemicellulose (%)	Lignin (%)
*Sargassum horneri*	28.29–39.88%	16.75–22.64%	22.10–27.20%
Eucalyptus [48]	37–46.9%	/	/
Corn fiber [49]	2.26–9.1%	36.4–46.4%	/
Corn stalk [50]	30.6–33.1%	25.8–27.65%	14.6–15.9%
Miscanthus sinensis [31]	40–60%	20–40%	10–25%
Big bluestem [43]	29.59–43.02	20.73–30.84	/
Moso bamboo [51]	37.98–53.76%	17.7–28.18%	13.82–23.86%

**Table 3:** Performance of the full-PLSR models with different pretreatment methods.

Method	Calibration				Prediction		
	$R_{C}^{2}$ ^1^	RMSEC ^2^ (%)	$R_{CV}^{2}$ ^3^	RMSECV ^4^ (%)	$R_{P}^{2}$ ^5^	RMSEP ^6^ (%)	RPD ^7^
Cellulose							
SG	0.9825	0.3274	0.5347	1.3672	0.6161	1.3833	1.6139
SG+1st	0.9998	0.0287	0.4955	1.4034	0.3407	1.8667	1.2316
SG+2nd	0.9942	0.1872	0.4440	1.4093	0.4802	1.6271	1.3871
SNV	1.0000	0.0033	0.2490	1.5854	0.3459	1.7723	1.2364
MSC	1.0000	0.0030	0.2495	1.5803	0.3479	1.7693	1.2383
Hemicellulose							
SG	0.8843	0.4579	0.6163	0.9491	0.5515	1.0277	1.4931
SG+1st	0.9998	0.0163	0.6132	0.9238	0.5558	0.9735	1.5004
SG+2nd	0.9696	0.2348	0.5681	0.9721	0.4724	0.9888	1.3767
SNV	1.0000	0.0024	0.5526	1.0135	0.3894	0.9823	1.2797
MSC	1.0000	0.0026	0.5483	1.0131	0.3787	0.9988	1.2687
Lignin							
SG	0.9467	0.2849	0.2382	1.5341	0.1935	1.5654	1.1135
SG+1st	0.9561	0.2589	0.1892	1.5265	0.1413	1.5676	1.0791
SG+2nd	0.8163	0.5292	0.2113	1.3426	0.2058	1.3833	1.1221
SNV	0.9759	0.1918	0.1259	1.5004	0.1025	1.5259	1.0556
MSC	0.9598	0.2478	0.1932	1.4760	0.1385	1.5947	1.0774

^1^ Coefficient of determination for calibration set; ^2^ root mean square error for calibration set; ^3^ coefficient of determination for cross-validation set; ^4^ root mean square error for leave-one-out cross-validation set; ^5^ coefficient of determination for prediction set; ^6^ root mean square error for prediction set; ^7^ residual predictive deviation.

**Table 4:** Performance of the full-PLSR models with different pretreatment methods.

Model		Calibration				Prediction		
	CVs ^1^	$R_{C}^{2}$	RMSEC (%)	$R_{CV}^{2}$	RMSECV (%)	$R_{P}^{2}$	RMSEP (%)	RPD
Cellulose								
Full-PLSR	8298	0.9825	0.3274	0.5347	1.3672	0.6161	1.3833	1.6139
iPLS-PLSR	1540	0.9827	0.3247	0.8511	0.7624	0.8955	0.8232	3.0934
CARS-PLSR	3261	0.9736	0.4017	0.6910	1.1304	0.7742	1.0637	2.1043
CC-PLSR	2485	0.9990	0.0783	0.7405	1.1877	0.7353	1.2988	1.9437
GA-PLSR	421	0.9339	0.6350	0.6808	1.1267	0.7418	1.1506	1.9680
Hemicellulose								
Full-PLSR	8298	0.9998	0.0163	0.6132	0.9238	0.5558	0.9735	1.5004
iPLS-PLSR	1935	0.9209	0.3786	0.8947	0.4983	0.8669	0.4697	2.7406
CARS-PLSR	6461	0.9998	0.0170	0.7581	0.8095	0.6962	0.9624	1.8143
CC-PLSR	1705	0.9723	0.2242	0.7661	0.7351	0.6746	0.7689	1.7532
GA-PLSR	731	0.9904	0.1320	0.7639	0.8181	0.7201	0.8029	1.8902
Lignin								
Full-PLSR	8298	0.8163	0.5292	0.2113	1.3426	0.2058	1.3833	1.1221
iPLS-PLSR	1665	0.9315	0.3232	0.8261	0.5172	0.7307	0.7533	1.9272
CARS-PLSR	3328	0.9726	0.2043	0.4423	0.9033	0.4119	0.9015	1.3040
CC-PLSR	2264	0.9411	0.2996	0.4139	1.3251	0.4460	1.1685	1.3435
GA-PLSR	899	0.8495	0.4789	0.5992	1.3164	0.3660	1.3635	1.5796

^1^ Characteristic variables.

**Table 5:** The results of the near-infrared spectroscopy measurement of terrestrial biomass and *Sargassum horneri*.

	Content	R^2^	RMSE	SEP
*Sargassum horneri*	Cellulose, hemicellulose and lignin	0.8955, 0.8669, and 0.7307	0.8232, 0.4697, and 0.7533	3.0934, 2.7406, and 1.9272
Eucalyptus [48]	Cellulose	0.82–0.94	0.7–1.07	/
Corn fiber [49]	Cellulose and hemicellulose	0.81–0.96 and 0.31–0.81	0.30–0.68 and 0.79–1.04	/
Miscanthus sinensis [31]	Cellulose, hemicellulose and lignin	0.943, 0.938, and 0.864	0.678, 0.707, and 0.562	/
Big bluestem [43]	Cellulose and hemicellulose	0.92 and 0.91	0.67 and 0.72	4.52 and 3.12
Moso bamboo [51]	Cellulose, hemicellulose and lignin	0.909. 0.921, and 0.892	0.81, 1.05, and 0.65	5.42, 3.18, and 1.62

## Data Availability

Not applicable.

## Sample Availability

Samples of the compounds are available from the authors.
